# Exposure–response relationship of sintilimab in advanced gastric cancer: insights from time-dependent pharmacokinetics and biomarker integration for precision immunotherapy

**DOI:** 10.3389/fphar.2026.1875485

**Published:** 2026-09-03

**Authors:** Jingyi Zhou, Jingzhi Wang, Lin Liu

**Affiliations:** 1 Department of Oncology, Zhongda Hospital, Medical School of Southeast University, Nanjing, Jiangsu, China; 2 Department of Radiotherapy Oncology, The First People’s Hospital of Yancheng, Affiliated Hospital of Medical School, Nanjing University, Yancheng, Jiangsu, China

**Keywords:** advanced gastric cancer, biomarkers, exposure-response relationship, immune checkpoint inhibitors, model-informed precision dosing, pharmacokinetics, sintilimab, time-dependent clearance

## Abstract

**Background:**

Immune checkpoint inhibitors (ICIs), particularly programmed cell death protein-1 (PD-1) antibodies, exhibit substantial interpatient variability in both efficacy and immune-related adverse events (irAEs). Conventional exposure-response (E-R) paradigms are often insufficient to capture these dynamics, limiting their utility for individualized immunotherapy.

**Objective:**

To critically re-evaluate the E-R relationship of Sintilimab in advanced gastric cancer (AGC) by integrating pharmacokinetics (PK), host- and tumor-related determinants, and multidimensional biomarkers.

**Methods:**

This review examines and critically discusses published PK and E-R data on Sintilimab and other PD-1/PD-L1 inhibitors, with a focus on time-dependent clearance, target-mediated drug disposition (TMDD), and inter-individual variability. Clinical and mechanistic studies linking exposure metrics, clearance (CL), efficacy, and irAEs were critically analyzed, and key covariates influencing drug exposure were identified.

**Results:**

Time-dependent CL confounds traditional exposure metrics. CL may serve as a surrogate marker reflecting host physiology, tumor burden, and systemic inflammation. Multi-level biomarkers modulate the translation of drug exposure into therapeutic efficacy and toxicity. Conceptually, the exposure–efficacy relationship of PD-1 inhibitors is more likely to be saturable or plateau-like after sufficient target occupancy, whereas higher exposure levels may increase the risk of irAEs without substantially enhancing efficacy beyond target saturation. Thus, the concept of an optimal benefit-risk range, rather than a simple “higher-exposure-is-better” strategy, is more appropriate for describing the E-R relationship of PD-1 inhibitors.

**Conclusion:**

We conceptualize a dynamic exposure–response framework for Sintilimab in AGC by integrating time-dependent clearance, disease-state confounding, biomarker modulation, therapeutic drug monitoring (TDM), and model-informed precision dosing (MIPD). This framework provides a conceptual basis for optimizing individualized therapy and improving the benefit-risk balance in patients with AGC.

## 1 Introduction

Patients with advanced gastric cancer generally have a poor prognosis. Despite ongoing optimization of chemotherapy and targeted therapy strategies in recent years, long-term survival benefits remain limited for most patients (Lin et al., 2024a). ICIs, particularly PD-1 antibodies, have emerged as a transformative therapeutic option, gradually becoming an important first-line strategy for AGC (Peng et al., 2024; Xu et al., 2023). Sintilimab, a PD-1 monoclonal antibody independently developed in China, has demonstrated favorable antitumor activity and manageable safety in multiple clinical studies (Lin et al., 2024a; Xu et al., 2023; Sun et al., 2024). However, in real-world practice, patient responses to Sintilimab are highly heterogeneous. Some patients achieve long-lasting remission, whereas others show limited efficacy or may experience severe irAEs (Mao et al., 2024). These observations suggest that fixed-dose administration may not fully reflect inter-individual differences in drug exposure and its impact on immune response, limiting treatment optimization.

To address this heterogeneity, the E-R relationship has emerged as a key theoretical framework for precision immunotherapy. ICIs exhibit unique PK characteristics, including TMDD and time-dependent CL (Centanni et al., 2019). Previous studies indicate that the pharmacokinetics of PD-1 inhibitors are closely associated with both clinical efficacy and irAEs (Garon et al., 2015). Evidence increasingly suggests that, as tumor burden and inflammatory status change during treatment, drug CL may dynamically decrease, resulting in elevated late-stage drug exposure (Wilkins et al., 2019). Conceptually, this bidirectional mechanism, in which efficacy may inversely affect PK, can produce apparent correlations rather than true causal relationships between exposure and efficacy, challenging traditional unidirectional E-R models. Further studies have shown that CL is not only a key PK parameter but may also serve as a surrogate indicator of patient disease state and prognosis (Maritaz et al., 2025; Otten et al., 2023; Guo et al., 2024). In contrast, correlations between conventional exposure metrics (e.g., area under the curve [AUC] or trough concentration [Ctrough]) and clinical efficacy are weak, suggesting that classic “exposure-driven efficacy” models may not adequately describe ICI treatment.

The factors influencing the drug exposure of sintilimab are complex and diverse, including physiological and pathological factors (such as body weight, albumin level, inflammatory status, and tumor burden), potential drug-drug interactions, and immunogenic factors (such as the production of anti-drug antibody, ADA) (Wu and Shao, 2025). Additionally, multi-level biomarkers, such as programmed death-ligand 1 (PD-L1) expression, tumor mutational burden (TMB), and microsatellite instability-high (MSI-H) or deficient mismatch repair (dMMR) further modulate the translation of drug exposure into therapeutic efficacy and toxicity (Holder et al., 2024). However, comprehensive investigations on the E-R relationship of Sintilimab in AGC, particularly in real-world populations, remain limited, and a unified analysis framework integrating PK and multi-dimensional biomarkers is lacking. The present review is a narrative review that critically discusses published evidence on the pharmacokinetics, exposure-response relationships, and biomarker integration of Sintilimab and other PD-1/PD-L1 inhibitors, with a focus on their relevance to AGC.

Herein, this review critically examines the E-R relationship of Sintilimab in AGC. Based on the synthesis of these multifaceted determinants, we conceptualize a dynamic exposure–response framework for sintilimab in AGC that integrates time-dependent clearance, disease-state confounding, biomarker modulation, TDM, and MIPD. This framework aims to provide a conceptual basis for optimizing individualized therapy and improving the benefit-risk balance in patients with AGC.

## 2 Pharmacokinetic characteristics of sintilimab: from classic parameters to time-dependent changes

### 2.1 Classical pharmacokinetic parameters and *in vivo* distribution characteristics

Sintilimab (IBI308) is a fully human IgG4 anti-PD-1 monoclonal antibody. Its PK characteristics generally follow the typical pattern of ICIs: affected by TMDD at low exposure, but exhibiting approximately linear CL and a relatively long elimination half-life (t1/2) within clinically recommended doses. IgG4 antibodies mainly restore T-cell function via PD-1 blockade; the Fc fragment minimally mediates antibody-dependent cellular cytotoxicity (ADCC) or complement-dependent cytotoxicity (CDC) (Kretschmer et al., 2017). This structural feature contrasts with IgG1 antibodies (e.g., Avelumab, Ipilimumab), which mediate additional effector functions via Fcγ receptors (Kretschmer et al., 2017; Beers et al., 2016).

Key PK parameters of Sintilimab include volume of distribution (Vd), CL, and t1/2. Vd is relatively small, primarily in plasma and extracellular fluid, indicating limited tissue penetration; the drug mainly targets immune cells in circulation and lymphoid tissues (Zhang et al., 2020). Due to structural similarity to Nivolumab and Pembrolizumab, Sintilimab PK parameters are generally comparable. For example, Nivolumab shows linear clearance (0.3–10 mg/kg), with CL ≈ 0.360 L/day (80 kg normalized), inter-individual variability ≈35%, t1/2 ≈ 25 days, and reaches steady state at ∼12 weeks (Fulchiero and Jimeno, 2014; Zhang et al., 2019). Pembrolizumab similarly exhibits linear clearance ≥0.3 mg/kg, with CL ≈ 0.168–0.249 L/day, inter-individual variability 30%–38%, and t1/2 14–27.3 days (Elassaiss-Schaap et al., 2017; Li et al., 2017; Ahamadi et al., 2017). Clinical PK studies of Sintilimab in advanced solid tumors indicate approximately linear kinetics between 1–10 mg/kg, with a single-dose elimination half-life of 14.4 days (coefficient of variation [CV] 28.9%), clearance of 11.5 mL/h (CV 42.5%), and volume of distribution of 5.43 L (CV 34.4%; apparent Vd 5.77 L, CV 33.2%). CL is relatively low among antibodies, likely due to FcRn-mediated recycling, which reduces lysosomal degradation and prolongs half-life, supporting a dosing regimen of every 2–3 weeks (Centanni et al., 2019; Keizer et al., 2010).

Drug exposure is commonly assessed by AUC and the steady-state trough concentration (Ctrough,ss). AUC quantifies overall exposure, which is influenced by Vd, CL, and t1/2. Systemic CL reflects all elimination pathways including TMDD, non-specific proteolysis, and ADA-mediated accelerated clearance. Ctrough,ss represents the lowest plasma concentration at the end of a dosing interval, closely related to continuous PD-1 receptor occupancy and essential for sustaining antitumor immune activity.

### 2.2 Target-mediated drug disposition and non-linear pharmacokinetic characteristics

The *in vivo* disposition of ICIs is primarily determined by two mechanisms. First, non-specific proteolytic degradation, which refers to the slow catabolism of antibodies via the reticuloendothelial system (RES) and the lysosomal pathway. Second, TMDD, a process of specific receptor-mediated endocytosis and degradation triggered by the binding of the drug to its pharmacological target. Meanwhile, the recycling mechanism mediated by FcRn (neonatal Fc receptor) re-releases internalized antibodies into the circulatory system, avoiding lysosomal degradation and prolonging t1/2 (Keizer et al., 2010).

TMDD is a key PK characteristic of monoclonal antibodies, in which high-affinity binding between the drug and target significantly affects distribution and elimination (Dua et al., 2015; Ryman and Meibohm, 2017). For PD-1 inhibitors, after binding to PD-1 receptors on T cells, drug-receptor complexes undergo receptor-mediated endocytosis and are subsequently degraded in lysosomes, forming a saturable clearance pathway (Dua et al., 2015). This process is regulated by factors such as target expression level, binding affinity, endocytosis rate, and intracellular degradation rate (Ryman and Meibohm, 2017).

**FIGURE 1:**
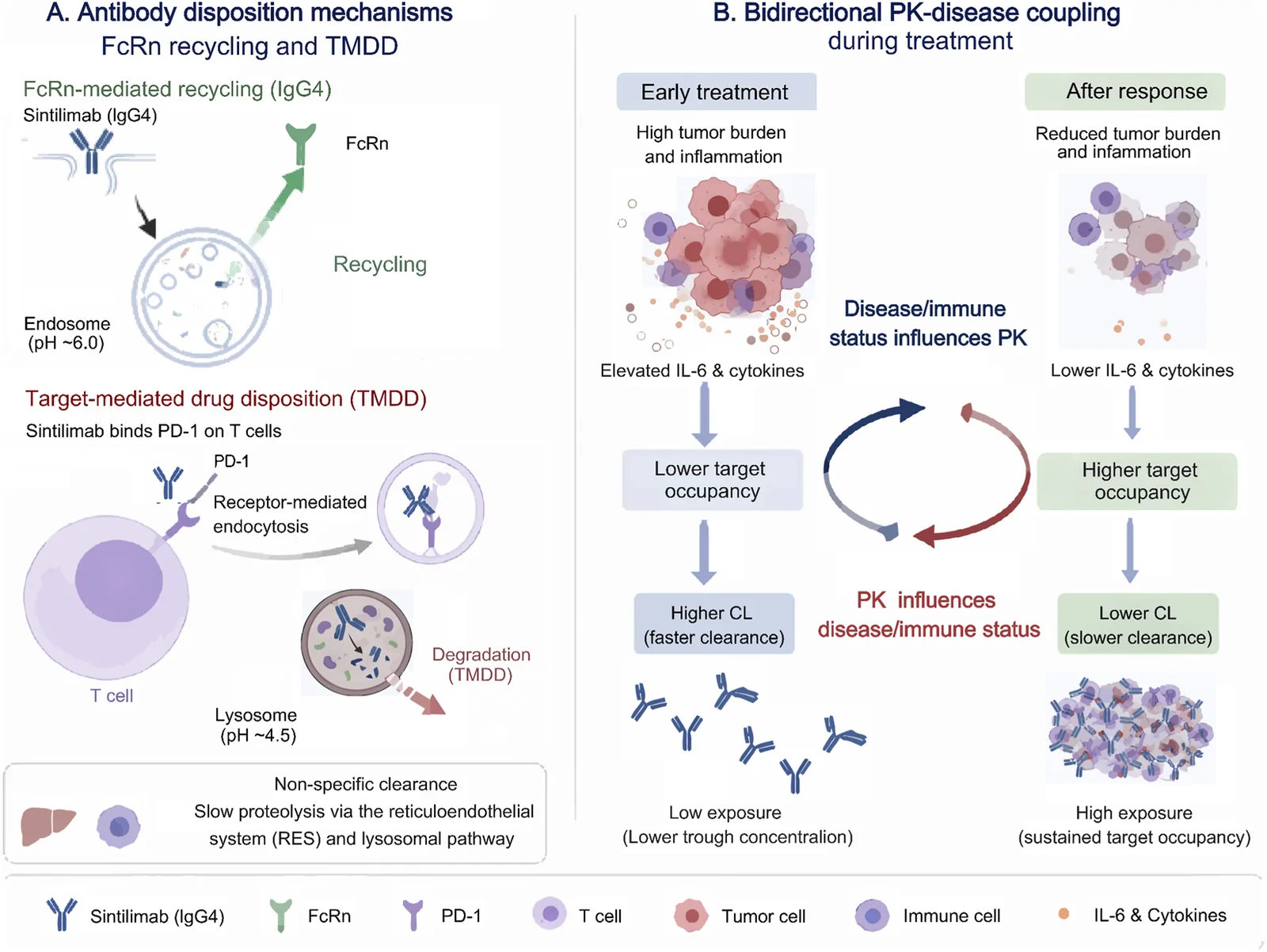
Conceptual overview of Sintilimab pharmacokinetics. Panel **(A)** (Antibody disposition mechanisms): FcRn-mediated recycling and target-mediated drug disposition (TMDD) illustrate antibody disposition. TMDD occurs when Sintilimab binds PD-1 on T cells, followed by receptor-mediated endocytosis → early endosome (pH ∼6.0) → lysosomal degradation; a portion of IgG4 antibodies is rescued via FcRn-mediated recycling. Non-specific clearance via the RES contributes to overall elimination. Panel **(B)** (Bidirectional PK–disease coupling): Baseline: low drug exposure leads to partial PD-1 occupancy; high tumor burden and elevated cytokines (e.g., IL-6) are associated with higher clearance (fast CL). Post-treatment: high drug exposure results in sustained PD-1 target occupancy; CL decreases due to FcRn recycling, reduced cytokines, and residual TMDD. Bidirectional arrows indicate the dynamic feedback loop between PK exposure and disease/immune status, highlighting how exposure modulates efficacy and toxicity. Annotations: Low exposure/high exposure, partial vs. full PD-1 receptor occupancy. Tumor cells (red), T cells and immune cells (purple), IL-6 and cytokines (orange), Sintilimab IgG4 (blue), FcRn (green), and PD-1 (purple) are indicated.

As illustrated in Figure 1, Sintilimab pharmacokinetics conceptually involves antibody disposition mechanisms, including FcRn-mediated recycling and TMDD on T cells, as well as bidirectional PK–disease coupling during treatment.

Within low-exposure ranges, PD-1 inhibitors are primarily affected by TMDD: after binding to PD-1, receptor-mediated endocytosis and degradation result in relatively fast CL. As PD-1 receptors become saturated, drug clearance increasingly relies on FcRn-mediated recycling and slow non-specific proteolysis via the RES, leading to overall non-linear PK characteristics (Dua et al., 2015; Liu, 2018). This shift from ‘receptor-mediated clearance’ to ‘non-specific clearance’ constitutes the core mechanism underlying the non-linear PK of PD-1 inhibitors.

Although TMDD significantly impacts the PK of monoclonal antibodies mechanistically, within clinically recommended doses of most PD-1 inhibitors (including Sintilimab), target saturation limits TMDD, and PK approximates linearity (Bajaj et al., 2017a). Therefore, TMDD primarily affects drug clearance at low exposure or during dose exploration phases.

TMDD also establishes a mechanistic link between PK and pharmacodynamics (PD) by regulating target occupancy and its duration, potentially influencing efficacy and the E-R relationship (Ryman and Meibohm, 2017). A thorough understanding of TMDD is essential for correctly interpreting the non-linear PK characteristics of ICIs, avoiding misjudgment of the E-R relationship, and optimizing dose design.

### 2.3 Dynamic changes in time-dependent clearance and its biological basis

It is worth emphasizing that time-dependent CL is an important characteristic of PD-1 inhibitors. For example, Nivolumab CL decreases by approximately 24.5% during treatment (Fulchiero and Jimeno, 2014) and Pembrolizumab CL decreases on average by 20%–30% over 10 months (Elassaiss-Schaap et al., 2017; Li et al., 2017; Ahamadi et al., 2017). This phenomenon is generally attributed to reductions in tumor burden and improvements in systemic inflammation (Li et al., 2017; Liu et al., 2017). Population PK studies indicate that Nivolumab CL is influenced by baseline physical status, body weight, estimated glomerular filtration rate (eGFR), race, and gender (Zhang et al., 2019). Pembrolizumab CL is affected by albumin, bilirubin, cancer type, and Eastern Cooperative Oncology Group performance status (ECOG-PS) (Ahamadi et al., 2017). Additionally, immunogenic factors can modulate the *in vivo* disposition of anti-PD-1 antibodies; ADA formation promotes endocytic uptake and degradation of ICIs, thereby increasing CL (Centanni et al., 2019).

Time-dependent CL, defined as changes in CL during treatment, is conceptually associated with optimal ORR (Li et al., 2017; Liu et al., 2017). A decrease in CL leads to an increase in Ctrough,ss (Zhao et al., 2023), with the magnitude correlating to disease state and clinical outcomes, which is critical for understanding exposure–response relationships (Liu et al., 2017). This pharmacokinetic characteristic may statistically create an illusion of “the higher the exposure, the better the efficacy” (Centanni et al., 2019; van Brummelen et al., 2016). If time-dependent CL is not fully considered in E-R analyses, observed associations between exposure and efficacy may be apparent rather than causal.

Currently, population PK studies on Sintilimab remain limited. Existing data indicate that inter-individual differences in CL are a major source of variability in drug exposure. Although systematic quantitative research on its time-dependent CL is lacking, mechanistic extrapolation suggests that CL changes dynamically with treatment, tumor burden, and inflammatory status, similar to other PD-1 inhibitors. Future research should focus on dynamic modeling of time-dependent clearance to more accurately explain the exposure-efficacy relationship of sintilimab and promote the optimization of individualized treatment strategies.

## 3 Determinants of drug exposure variability: a three-dimensional framework of host-tumor-immune system

### 3.1 Host factors: demographic characteristics, nutritional status, and inflammation level

Clinical practice indicates that PD-1 inhibitors can reach a steady-state plasma concentration in patients after multiple regular administrations. However, despite the widespread use of fixed-dose administration regimens in clinical practice, there are still significant differences in the steady-state plasma concentrations among individuals, especially the steady-state trough concentration (Ctrough,ss) at the end of the dosing interval, with the amplitude reaching several times (Centanni et al., 2019; Elassaiss-Schaap et al., 2017; Bajaj et al., 2017a). During the treatment process, the clearance rate of PD-1 inhibitors decreases dynamically as the tumor burden decreases and inflammation improves, which affects the steady-state plasma concentration and the exposure-response relationship (Elassaiss-Schaap et al., 2017; Li et al., 2017; Ahamadi et al., 2017; Liu et al., 2017). Based on the discovery of time-dependent clearance, population pharmacokinetic (PopPK) models that include time-varying clearance rates have been developed for ICIs. Key covariates focus on the patient’s baseline demographic characteristics and pathophysiological states, such as body weight, body surface area (BSA), race, gender, ECOG-PS, cancer type, maximum tumor diameter, the sum of longest diameters (SLD) of target lesions, eGFR, serum albumin, bilirubin level, lactate dehydrogenase (LDH), lymphocyte count, neutrophil count, and alkaline phosphatase. These factors are commonly recognized to affect the CL of ICIs and contribute to inter-individual differences in their plasma concentrations (Wilkins et al., 2019; Zhao et al., 2023; Bajaj et al., 2019; Marchand et al., 2021).

Population pharmacokinetic studies have shown that in the Avelumab study targeting 14 different cancer types, only Merkel cell carcinoma (mMCC) and squamous cell carcinoma of the head and neck (SCCHN) exhibited a significant decrease in clearance. The clearance of the former was maximally reduced by 32.1% compared to the baseline, and that of the latter was maximally reduced by 24.7%. These results indicate that the time-dependent change in clearance is closely related to treatment response, with a more significant decrease observed in responders (Wilkins et al., 2019). In addition, the CL of Nivolumab is affected by factors such as ECOG-PS, body weight, eGFR, race, and gender (Fulchiero and Jimeno, 2014), while the CL of Pembrolizumab is influenced by albumin, bilirubin, cancer type, and ECOG-PS (Ahamadi et al., 2017). The CL of Atezolizumab is significantly affected by body weight, albumin, ADA, gender, neutrophil count, alkaline phosphatase, bilirubin level, and SLD (Stroh et al., 2017). Based on the data from the phase II/III clinical trials of non-small cell lung cancer (NSCLC) patients treated with Pembrolizumab, the PopPK model shows that the time-dependent clearance of Pembrolizumab is related to the sum of the SLD, LDH, lymphocyte count, and albumin level (Li et al., 2019). The above studies suggest that patients with low body weight and low albumin levels usually exhibit higher drug CL. This is generally consistent with the previous understanding that nutritional status, inflammation level, and serum protein metabolism affect pharmacokinetic behavior (Xu et al., 2022), and also aligns with the finding that low baseline albumin level indicates poor prognosis for ICI treatment (Yao et al., 2025). Hypoalbuminemia (usually defined as < 35 g/L) is common in patients with AGC and cachexia. The time-dependent CL characteristics of ICIs may be partly due to their indirect effect of reversing cancer-related cachexia and improving the overall disease state (Zhao et al., 2023). Cachexia is a metabolic syndrome associated with the underlying disease, often accompanied by hypercatabolism and muscle mass loss. This metabolic abnormality can promote the degradation of endogenous proteins and therapeutic monoclonal antibodies (Ryman and Meibohm, 2017; McMillan, 2013). Therefore, in patients with advanced cancer, cachexia often accelerates the clearance of proteins and drugs. As treatment takes effect, the patient’s catabolism is alleviated, protein synthesis is restored, and the clearance rate can gradually decrease, manifested as an increase in steady-state plasma drug concentration, an effect that is particularly pronounced in patients with significant treatment response (Zhao et al., 2023; Liu et al., 2021a).

In conclusion, the time-dependent CL of ICIs is closely related to patients’ baseline characteristics and disease status. These factors jointly influence individual differences in drug clearance and treatment outcomes. The establishment of PopPK models provides an important tool for further understanding the relationship between tumor burden, inflammatory status, and the PK of ICIs.

### 3.2 Tumor factors: tumor burden and regulation of the tumor microenvironment

Studies have shown that the time-varying CL characteristics of ICIs may stem not only from their indirect effects of reversing cancer-related cachexia and improving disease state but also from tumor-driven changes in drug consumption. Tumors may participate in drug elimination through local metabolism or drug retention in tumor tissues (Zhao et al., 2023).

The immune cells, blood vessels, and stromal components in the tumor immune microenvironment (TME) collectively shape the immune characteristics of tumors, thereby exerting a significant impact on the PK and efficacy of ICIs. Tumor size, type, and burden, as well as the metabolic state of the TME, may influence treatment outcomes by directly or indirectly affecting drug clearance and immune response (Racacho et al., 2025). A retrospective study on AGC showed that among patients receiving neoadjuvant therapy with sintilimab combined with the SOX regimen (oxaliplatin + tegafur-gimeracil-oteracil potassium), a tumor diameter >2 cm was an independent risk factor for shorter progression free survival (PFS) (Wang et al., 2025). Another study on mid-to-advanced rectal cancer found that the larger the tumor diameter, the worse the efficacy of sintilimab combined with neoadjuvant chemotherapy (Wang et al., 2022a). These findings suggest that a larger tumor volume not only represents advanced disease but may also form a ‘drug consumption pool’, reducing effective drug exposure. Population pharmacokinetic studies have shown that during immunotherapy with ICIs such as Pembrolizumab (Li et al., 2019), Atezolizumab (Stroh et al., 2017), and Avelumab (Wilkins, 2017), SLD, cancer type, and tumor burden can cause variations in CL. Tumor size evaluated by imaging and the number of metastatic lesions are positively correlated with drug CL, and CL decreases when tumor burden is reduced (Zhao et al., 2023). High tumor burden may increase drug consumption through the TMDD mechanism. The core of TMDD lies in the availability of cell-surface targets. Whether targets are located on tumor cells or on T cells that respond to tumor cells, both are related to drug clearance. When tumor burden is high, more PD-1 receptors appear on the surface of tumor cells and immune cells in the TME, increasing drug binding and endocytic clearance; when burden is reduced, the number of surface targets decreases, thus limiting the degradation of PD-1 inhibitors (Centanni et al., 2019). In the TME, hypoxia is a common metabolic feature of solid tumors, associated with abnormal angiogenesis, increased tumor interstitial pressure, and metabolic reprogramming (Bigo et al., 2024). Hypoxia affects drug delivery by altering tumor blood flow and tissue physical properties (e.g., permeability). Abnormal blood vessels and increased interstitial pressure can also reduce the effective concentration of drugs reaching tumor cells, representing a potential mechanism for uneven distribution, phagocytic clearance, and drug resistance of macromolecular drugs (Roy et al., 2020). Systemic inflammatory status is another important feature of the TME. Elevated systemic inflammatory markers not only reflect disease activity but are also closely associated with accelerated clearance and reduced efficacy of ICIs. The neutrophil-to-lymphocyte ratio (NLR) is a commonly used indicator for assessing systemic inflammation. A high NLR is usually associated with an immunosuppressive TME and poor prognosis. Studies have shown that NLR can predict the efficacy of Sintilimab (Wang et al., 2022a; Tao et al., 2024); the dynamic change in NLR (ΔNLR%) during treatment has stronger predictive value (Tao et al., 2024). The underlying mechanisms include: inflammatory factors such as IL-6 upregulate the expression of FcRn in the liver, accelerating the decomposition of IgG antibodies; inflammation is accompanied by hypermetabolism and changes in vascular permeability, affecting the volume of distribution and clearance rate (Seo et al., 2022); a high NLR indicates an increase in myeloid-derived suppressor cells (MDSCs), weakening the anti-tumor effect of T cells (Tao et al., 2024).

In summary, immune cells, tumor burden, and metabolic status in the TME influence the PK and efficacy of ICIs, providing guidance for individualized treatment strategies.

### 3.3 Immunity-related factors: immunogenicity and genetic background

Pharmacogenetics and immunogenicity indicators are also important biological factors determining the PK variability of ICIs and may further affect efficacy and safety. Beyond host and tumor characteristics, the PK of ICIs, as well as inter- and intra-patient variability, is additionally regulated by immunity-related factors including ADA formation, changes in proteolytic function, and FcRn genetic polymorphisms (Centanni et al., 2019).

The CL of ICIs can occur through degradation pathways mediated by humoral and cellular immunity. The formation of ADA facilitates the uptake and endocytic degradation of ICIs, thereby increasing CL (Centanni et al., 2019), and is a key indicator for predicting declining drug concentrations and potential treatment failure. ADA can bind to antibodies to form complexes, accelerating clearance or neutralizing pharmacological activity, leading to decreased drug concentrations and loss of treatment response (Ortiz-Fernández et al., 2025). For example, in adalimumab treatment, low trough drug concentration is significantly associated with the risk of treatment discontinuation (Ortiz-Fernández et al., 2025). This finding indicates that ADA production not only affects drug PK but may also alter its pharmacodynamic response. As a key immunogenic factor affecting ICI CL, ADA formation can increase the CL of anti-PD-1 antibodies, and its incidence is affected by drug type and combination therapy. Existing studies suggest that the incidence of ADA for Nivolumab is approximately 11.2%, which can increase CL by about 14%; the incidence of ADA for Pembrolizumab is approximately 0.7%–2.5% (Li et al., 2017; Bajaj et al., 2017a; Vugt et al., 2016). In the context of combination therapy, the impact of ADA on ICI PK is more significant than in monotherapy (van Brummelen et al., 2016). Among patients receiving combination therapy with Ipilimumab and Nivolumab, the proportion of patients producing ADA against Nivolumab increases significantly (approximately 10%–21.9%), accompanied by an approximately 24% increase in the CL of Nivolumab (Zhao et al., 2017). In contrast, for Sintilimab, a fully humanized antibody, the incidence of ADA and its impact on CL remain to be further clarified. The formation of ADA also depends on the structure of monoclonal antibodies. The immunogenicity of antibodies progressively increases from human (-umab: 0%–10%) to humanized (-zumab: 0.4%–18.5%) to chimeric (-ximab: 1%–17%) antibodies (van Brummelen et al., 2016), consistent with the conclusion that in most ICIs (whether human or humanized antibodies), the proportion of patients forming ADA and its impact on CL are considered limited (Centanni et al., 2019). In summary, ADA formation is an important source of individual differences in the CL of ICIs, and its incidence is closely related to the drug structure and combination therapy regimen.

Fcγ receptor (FcγR) gene polymorphisms indirectly regulate the PK and pharmacodynamic (PD) behaviors of ICIs by influencing the binding efficiency of drugs to the Fc segment on immune effector cells such as natural killer (NK) cells and macrophages (Ioannou et al., 2025). Fc-dependent effects, including ADCC, CDC, and antibody-dependent cellular phagocytosis (ADCP), are closely related to antibody subtypes (Centanni et al., 2019; Kretschmer et al., 2017). Compared with IgG4-class ICIs that mainly exert receptor-blocking effects, IgG1-class ICIs are more likely to mediate the clearance of regulatory T cells (Tregs) in tumors and induce stronger cellular and humoral immune effects (Centanni et al., 2019; Kretschmer et al., 2017; Beers et al., 2016). From a PK perspective, stronger Fc-mediated effects may be accompanied by more pronounced target cell clearance and drug consumption; IgG4-class ICIs have weaker binding to effector cells, lower CL, and longer half-lives, thus maintaining higher *in vivo* exposure levels.

Specific FcγR genotypes, such as the V158F polymorphism of the FCGR3A gene, have been confirmed to be associated with the clinical efficacy of other therapeutic antibodies, such as rituximab. In pharmacogenomic studies of inflammatory bowel disease, NUDT15 and TPMT variants vary significantly among different ancestral populations and directly affect drug response and toxicity (Ioannou et al., 2025). This suggests that in patients with AGC, FcγR polymorphism may affect Sintilimab CL and final blood drug concentration by altering its interaction with the immune system. However, in-depth studies on Sintilimab in this regard are still awaited.

Multiple genetic studies have shown that HLA class II allele polymorphism is closely associated with ADA formation. Patients carrying variants such as HLA-DQA1*05 have a significantly higher incidence of ADA during anti-tumor necrosis factor (anti-TNF) treatment and exhibit higher antibody titers and lower drug persistence, suggesting that HLA genotypes may serve as important genetic markers for ADA sensitivity (Sazonovs et al., 2020). Additionally, HLA loci such as HLA-DRB1*03 have also been found to be associated with ADA production in therapeutic antibodies, further supporting the influence of genetic background on immunogenic responses (Liu et al., 2018). Retrospective studies based on large-scale genetic analysis also suggest that HLA class II haplotypes can serve as potential genetic markers for predicting ADA sensitivity, indicating that HLA polymorphism is involved in regulating the immune system’s ability to recognize therapeutic antibodies (Mosch and Guchelaar, 2022). Therefore, as part of the patient’s immune responsiveness, HLA polymorphism may affect the immunogenicity and PK behavior of biological agents.

In summary, immunogenic reactions, especially ADA formation, are important factors influencing the PK and efficacy of ICIs. Evaluating the incidence of ADA and its impact on drug CL helps identify potential risks of decreased drug exposure and treatment failure, providing a scientific basis for clinical medication. Future research needs to further clarify the relationship between immunogenicity, genetic markers, and treatment response, and promote the integrated application of genomics and immunology information in personalized treatment.

**FIGURE 2:**
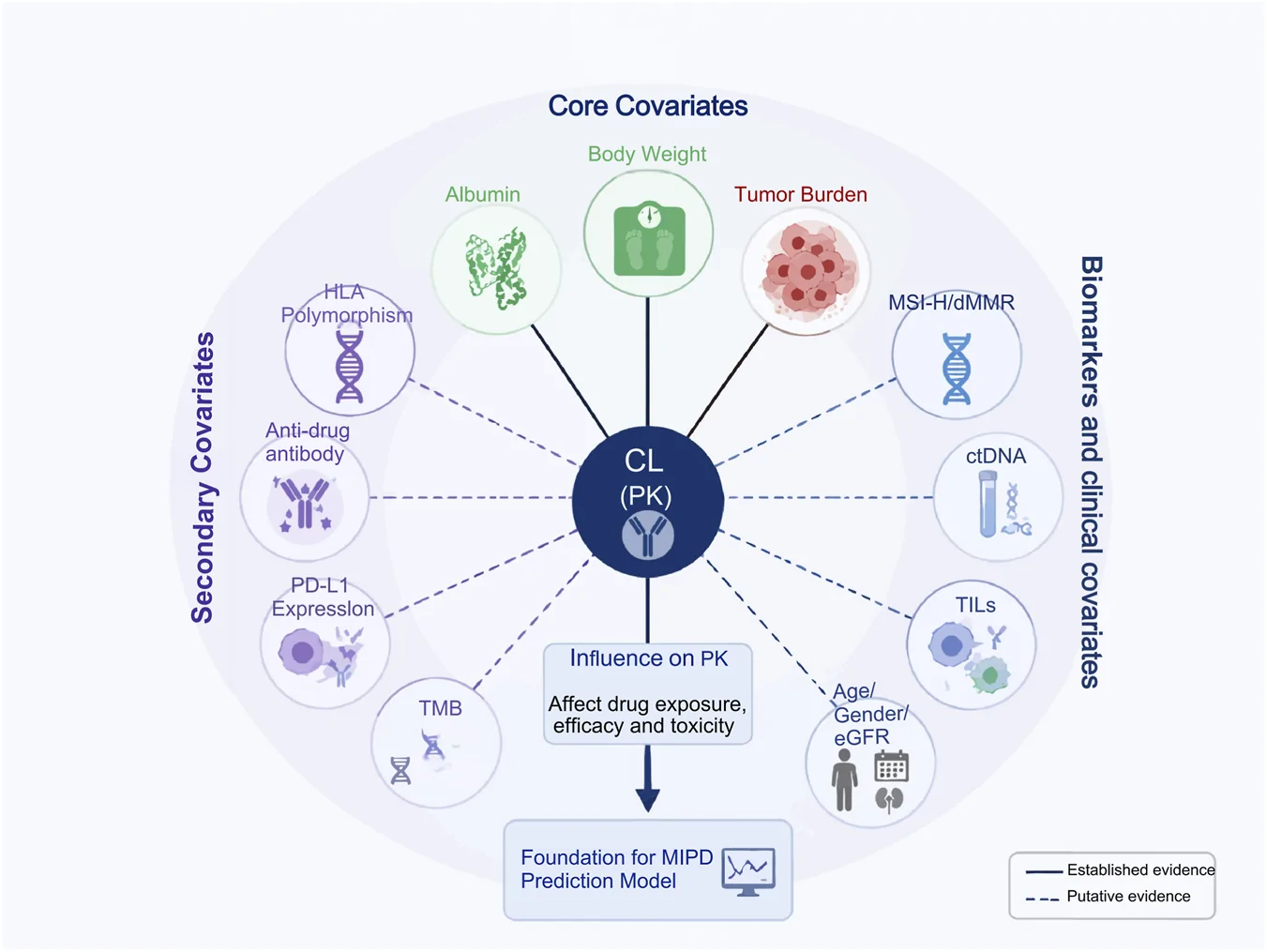
Covariates and Multi-dimensional Biomarker Matrix. Core covariates include Body Weight, Albumin, and Tumor Burden (established/major evidence). Secondary covariates/biomarkers include HLA polymorphism, ADA, PD-L1 expression, TMB, MSI-H/dMMR, ctDNA, TILs, and Age/Gender/eGFR (putative/emerging evidence). Solid lines indicate major influence on PK/CL, dashed lines indicate modest influence.

Figure 2 summarizes the key covariates and their relative impact on Sintilimab clearance.

## 4 Reexamination of the exposure-response relationship: from the traditional E-R to dynamic models

### 4.1 Biological basis of the exposure-response relationship: PK-PD coupling mechanism

In the pharmacokinetic-pharmacodynamic studies of ICIs, the relationship between drug exposure indicators (such as Ctrough, Cmin, and AUC) and clinical efficacy has always been an important research direction for dose optimization and individualized treatment. However, different from traditional cytotoxic drugs, the exposure-efficacy relationship of ICIs shows significant complexity, and different targets even exhibit different E-R patterns.

The inconsistency in the exposure-efficacy relationship of ICIs may be related to the target-binding characteristics and time-dependent clearance rate. In an early phase I study of Nivolumab, it was found that within the dose range of 0.1–10 mg/kg, the occupancy rate of PD-1 receptors on peripheral T cells remained at approximately 60%–70%, and there was no significant correlation with the dose. This indicates that sufficient target blockade can be achieved and a sustained immune activation state can be maintained at a relatively low dose level, thus forming a relatively flat PK-PD relationship (Liu et al., 2017). Similarly, research results have shown that within 4 weeks of intravenous infusion of Sintilimab, the continuous occupancy rate of PD-1 receptors exceeds 95%, suggesting that its higher affinity and unique PD-1 epitope may explain its favorable clinical efficacy (Wang J. et al., 2019). Therefore, during anti-tumor treatment with PD-1 inhibitors, once the saturation state of target occupancy is reached, further increasing drug exposure usually does not significantly enhance the immune activation effect, resulting in a plateau-like characteristic of the exposure-efficacy relationship. This mechanism explains the exposure-response pattern of ICIs, which is different from that of traditional drugs, at the biological level.

### 4.2 Methodological challenges: time-dependent clearance and reverse causation bias

Previous drug development experience suggests that clarifying the relationship between drug exposure and clinical response is of great significance for dose optimization, patient stratification, and regulatory decision-making. Traditional E-R analysis is usually based on the unidirectional causal hypothesis, i.e., drug exposure determines treatment response. However, for ICIs, this classic hypothesis is challenged because the clearance rate during treatment decreases dynamically with the reduction of tumor burden and the improvement of inflammation. Therefore, the interaction between treatment response and PK needs to be considered in exposure-efficacy analysis; otherwise, it may lead to biases in the E-R relationship (Zhao et al., 2023). A simulation study confirmed that compared with late-stage exposure indicators (such as average steady-state concentration, Cavg,ss) which may be interfered with by treatment outcomes, using early-stage exposure indicators (such as the average concentration after the first dose, Cavg1) may reduce the confounding caused by time-varying clearance rate and more accurately reflect the true contribution of exposure to efficacy (Liu et al., 2017). In addition, the imbalance of prognostic risk factors among different study groups may also interfere with the interpretation of the exposure-efficacy relationship. Therefore, in the E-R analysis of ICIs, early-stage exposure indicators should be preferentially used and potential prognostic factors should be fully corrected to obtain more reliable causal inferences (Zhao et al., 2023). Recent methodological work by Proctor and Wong (Proctor and Wong, 2024)and Yin et al. has further emphasized the importance of accounting for time-varying confounding and selecting appropriate exposure metrics in E-R analyses (Lin et al., 2024b).

In recent years, further research has indicated that during anti-tumor treatment with PD-1 inhibitors, CL may better reflect the patients’ disease status and prognosis than the blood drug concentration (Guo et al., 2024).

### 4.3 Clinical evidence and limitations of the exposure-efficacy relationship

Multiple clinical studies have confirmed that the time-varying clearance characteristics of ICIs lead to a complex E-R relationship. Studies by Bajaj, Turner, et al. have shown that the early exposure indicator Cavg1 of Nivolumab is not an independent predictor of OS, while the baseline CL is continuously and significantly associated with OS. After excluding the influence of baseline clearance in the sensitivity analysis, within the dose range of 1–10 mg/kg, the E-R relationship between the exposure level of Nivolumab and efficacy is relatively flat, suggesting a wide therapeutic window and no obvious dose-efficacy gradient (Bajaj et al., 2017b; Feng et al., 2017). Turner, Kondic, and others, based on the randomized trial data of melanoma and NSCLC, found that the OS improvement pattern of patients with high Nivolumab exposure is similar to that across the entire dose gradient. However, patients with the worst survival are concentrated in the highest quartile group of CL, indicating that the difference in outcomes is mainly driven by CL rather than the administered dose or exposure level (Turner et al., 2018). This finding supports the hypothesis that a higher clearance rate of ICIs may reflect a more severe disease state in patients, such as cachexia syndrome and systemic inflammatory response, which is associated with a poorer prognosis (Feng et al., 2017). Wang et al. used machine learning methods to construct a cytokine signature model to predict the baseline clearance of Nivolumab and found that patients with high CL have poor survival regardless of whether they receive Nivolumab or chemotherapy (Wang et al., 2020; Wang R. et al., 2019). Clearance rate is considered to have potential value for early prediction of efficacy, and relevant studies are being carried out for verification. At the first dose, the hazard ratio (HR = 3.55, 95% CI 1.75–7.20) of NSCLC patients with an initial Nivolumab clearance rate ≥7.3 mL/h is significantly higher than that of patients with a clearance rate <7.3 mL/h, suggesting that the clearance rate has potential value as a prognostic biomarker (Otten et al., 2023). Similar studies have also shown that patients with a Pembrolizumab clearance rate >0.232 L/day at the first administration are more likely to experience disease progression (HR = 1.98, 95% CI 1.21–3.26, *P* = 0.007) or have poor survival (HR = 2.04, 95% CI 1.16–3.59, *P* = 0.014), indicating that an increase in the clearance rate is significantly associated with disease progression and poor survival (de Vries et al., 2025). Based on the fact that a lower baseline clearance rate or a significant decrease in the clearance rate during treatment is usually associated with a better clinical response and longer survival, the clearance rate of antibody drugs may become one of the important indicators for evaluating the efficacy of immunotherapy (Guo et al., 2024).

Among different categories of ICIs, the E-R relationship of Cytotoxic T-lymphocyte associated antigen 4 (CTLA-4) inhibitors is relatively clear. Taking Ipilimumab as an example, three melanoma studies (NCT00135408, NCT00289627, and NCT00289640) included 419 patients. The steady-state minimum plasma concentration (Cmin,ss) of patients receiving different doses (0.3–10 mg/kg) was a significant predictor of the objective response rate (ORR), immune-related response rate (irRC), and OS (Centanni et al., 2019; Feng et al., 2013). The correlation with irRC was stronger than that with ORR, and the correlation between dose and OS was relatively weak (Sharma et al., 2023).

In contrast, the E-R relationship of PD-L1 inhibitors is weak and heterogeneous. For example, in the IMvigor210 study (NCT02108652) of Atezolizumab, an E-R analysis was conducted on 306 patients with urothelial cancer. The results showed that multiple exposure indicators such as area under the curve from day 0 to day 21 (AUC21), steady-state AUC (AUCss), maximum concentration (Cmax), and minimum concentration (Cmin) were not significantly correlated with ORR (Stroh et al., 2017). Similar results were also observed in the study of Durvalumab. In patients with urothelial cancer, Cmax, Cmin, and Cmin,ss did not show a clear relationship with the efficacy endpoints (Powles et al., 2017). However, in the phase I clinical trial of PCD4989g, an exploratory analysis of 88 patients with NSCLC who received Atezolizumab at a dose of 10–20 mg/kg by intravenous injection found that tumor shrinkage was significantly correlated with AUCss, baseline factors (metastatic sites, liver metastasis, and smoking status), and the relative change in interleukin (IL)-18 level on day 21 relative to the baseline. Moreover, AUC was the main predictor of ORR (Netterberg et al., 2019). In addition, in the study of Avelumab, although a certain correlation was observed between Ctrough,ss and progression-free survival (PFS) and OS, this association may be confounded by time-dependent clearance because treatment response can lead to a decrease in clearance rate and an increase in late drug exposure (Wilkins, 2017). Overall, these studies suggest that the exposure-efficacy relationship of PD-L1 inhibitors is relatively limited and the research results are somewhat heterogeneous. Recent PK-PD studies have also indicated that in PD-L1 antibody therapy, the inter-patient exposure variability usually exceeds the pharmacological threshold required for immune activation. Therefore, the impact of exposure level changes on efficacy is relatively limited (Yan et al., 2023).

Regarding PD-1 inhibitors, a consistent conclusion on the exposure-efficacy relationship has not yet been reached. In multiple studies of Nivolumab (NCT00730639, NCT01721746), patients with melanoma, NSCLC, and renal cell carcinoma were included, with the dose ranging from 0.1 to 10 mg/kg. The study results showed that in melanoma patients, no obvious relationship was observed between ORR and the dose, but there was a certain correlation between Cmin,ss and the ORR, and this relationship tended to reach a plateau when the dose was ≥1 mg/kg (Agrawal et al., 2016). In addition, no significant association was established between Cavg1 and the ORR or OS, while the survival outcome was negatively correlated with the drug clearance rate (Wang et al., 2017). A similar phenomenon was also observed in patients with NSCLC, where the relationship between Cmin,ss and the ORR tended to reach a plateau when the dose was ≥3 mg/kg (Agrawal et al., 2016). In the study of Pembrolizumab, the E-R analysis of patients with melanoma and NSCLC also did not show a significant relationship between the 6 -week AUC and the tumor shrinkage rate or ORR (Chatterjee et al., 2017; Chatterjee et al., 2016).

In summary, there is generally a relatively flat E-R relationship between drug exposure and efficacy during ICIs treatment. CL, as an important PK parameter reflecting the patient’s disease state, has a significant and stable association with clinical outcomes. Therefore, it is crucial to fully consider CL and its confounding effects on exposure indicators in E-R analysis, select appropriate exposure indicators, and incorporate key covariates such as baseline CL into the statistical model to avoid time-varying PK bias and accurately interpret the E-R relationship (Ruiz-Garcia et al., 2023; Petitcollin et al., 2020).

In addition, although previous studies have suggested that Sintilimab has a high PD-1 receptor occupancy rate and good efficacy, research on the exposure-efficacy relationship in real-world populations remains limited, and there is a lack of systematic analysis. This indicates an urgent need for systematic evaluation of its exposure–efficacy relationship to guide clinical application.

### 4.4 The regulatory role of biomarkers in the exposure-efficacy relationship

Notably, biomarkers are not only tools for predicting efficacy but also important regulatory links connecting drug exposure and immune effects, thus playing a crucial role as mediators in E-R relationship. The therapeutic effect of ICIs is not a simple linear relationship between drug exposure and tumor response but a dynamic process deeply regulated by a complex biomarker network. In this framework, biomarkers can serve as important covariates in the E-R model and reflect the intrinsic characteristics of tumors and hosts as well as the dynamic changes during treatment. According to their characteristics of action, they can be classified into three categories: static biomarkers, dynamic biomarkers, and individualized functional biomarkers. Static biomarkers include indicators that have been approved by the US Food and Drug Administration (FDA) or are widely concerned, such as PD-L1, MSI-H, and TMB. Dynamic biomarkers include circulating tumor DNA (ctDNA), gut microbiome, and radiomics. Individualized functional biomarkers include neoadjuvant treatment response and functional models such as patient-derived organoids or organ-on-a-chip (Holder et al., 2024).

Although static biomarkers such as PD-L1 expression, TMB, and MSI-H are only evaluated at a single time point, they set the foundation for efficacy potential before drug exposure. For example, tumors with high PD-L1 expression are more likely to relieve immune suppression after ICIs reach effective exposure levels, thereby enhancing the anti-tumor immune response (Holder et al., 2024). In NSCLC, patients with PD-L1 tumor proportion score (TPS) ≥ 50% showed significantly improved OS after using Pembrolizumab (Reck et al., 2016). The ORIENT-16 study also clearly showed that PD-L1 combined positive score (CPS) is an important predictor of the efficacy of Sintilimab (Xu et al., 2023; Xu et al., 2021). In China, based on the results of this trial, sintilimab in combination with chemotherapy has been approved as a first-line treatment for patients with advanced or metastatic gastric cancer who are HER2-negative and have PD-L1 CPS ≥5. This regulatory context is jurisdiction-specific and should not be extrapolated globally. To date, sintilimab has not received regulatory approval from the US FDA or the European Medicines Agency (EMA) for the treatment of gastric cancer. Similarly, tumors with high TMB and MSI-H can more effectively activate the immune response and amplify the efficacy under the same drug exposure conditions due to their high neoantigen load and immunogenicity (Marabelle et al., 2020; Ionov et al., 1993; Marcus et al., 2019). These baseline characteristics regulate the degree of immune system activation at the same drug exposure level, thus affecting clinical efficacy. However, their predictive power may still be affected by tumor heterogeneity, detection standardization, and other intrinsic tumor or host factors (such as POLE/POLD mutations) (Diaz et al., 2022; Heitzer and Tomlinson, 2014; Jansen et al., 2016; Albertson et al., 2009).

In contrast, dynamic biomarkers can provide more detailed real-time regulatory information on the exposure-efficacy relationship. By comparing biopsy samples before and after treatment, the impact of drug exposure on the TME can be directly revealed. For example, after ICIs treatment, an increase in tumor-infiltrating lymphocytes (TILs) or a decrease in immunosuppressive cells in the tumor microenvironment directly reflects the degree of immune system activation. This is a key intermediate link in the conversion of drug exposure into clinical efficacy, thus enabling earlier and more accurate identification of responders (Chen et al., 2016; Liu D. et al., 2021). A decrease in hematological biomarkers such as ctDNA levels indicates that drug exposure is effectively inhibiting tumor growth and reducing tumor burden (Zviran et al., 2020; Parikh et al., 2021). An increase in activated T cells or a decrease in exhausted CD8^+^ T cells in peripheral blood mononuclear cells (PBMCs) reflects the effect of drug exposure on activating the host immune system (Huang et al., 2017; Nixon et al., 2019; Du et al., 2018; Fairfax et al., 2020; Valpione et al., 2020). Radiomics reveals the macroscopic biological impact of drug exposure on tumors by quantifying changes in tumor volume and internal heterogeneity before and after treatment, and can assist in identifying pseudoprogression (Basler et al., 2020; Carvalho et al., 2016). In addition, the gut microbiome significantly affects the efficacy of ICIs by regulating the host immune system (Holder et al., 2024; Gopalakrishnan et al., 2018). The abundance of specific gut microbiota (such as bacteria in the Ruminococcaceae family or Akkermansia muciniphila) can significantly enhance or inhibit the anti-tumor response of the immune system under the same drug exposure (Derosa et al., 2020; Fang et al., 2022).

Individualized functional biomarkers further promote the transformation of the exposure-efficacy relationship towards precision medicine. Neoadjuvant therapy can directly evaluate the relationship between drug exposure and major pathological remission (MPR) to guide subsequent treatment (Marron et al., 2022; Mittendorf et al., 2022). Patient-derived tumor models such as tumor organoids and organ-on-a-chip can simulate the TME and immune interactions of patients *in vitro*, which are used to predict drug sensitivity at different exposure levels, thus providing a functional basis for individualized treatment (Hofmann et al., 2022; Wang et al., 2023; Leung et al., 2022). In addition, a combination therapy study showed that the clinical benefit of Sintilimab is associated with the regulation of the TGF-β/Smads pathway. The decrease of TGF-β1 and Smad2 and the increase of Smad7 are significantly associated with better survival (Holder et al., 2024; Hofmann et al., 2022), suggesting that the TGF-β pathway is a potential target for efficacy prediction and combination strategies.

In summary, biomarkers regulate the complex relationship between drug exposure and efficacy in multiple dimensions by integrating tumor and host characteristics, dynamic changes in treatment, and individual functional assessment. The future challenge lies in integrating multimodal information to construct more comprehensive and predictive algorithmic models, accurately identifying patients most likely to achieve long-term survival benefits, and guiding individualized immunotherapy strategies to realize precision immuno-oncology.

**FIGURE 3:**
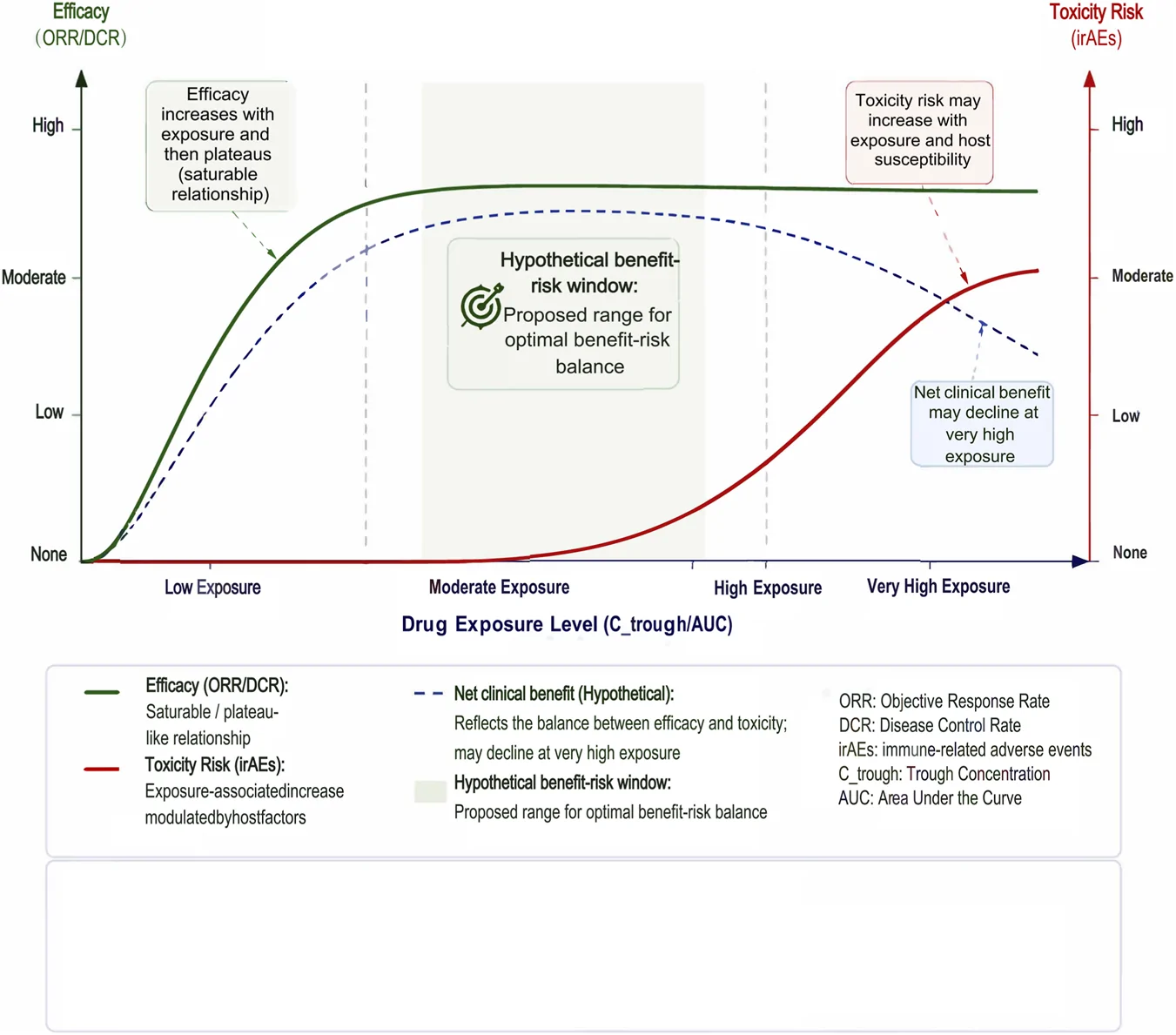
Hypothetical benefit–risk window for Sintilimab exposure (schematic illustration). The green curve depicts the relationship between Sintilimab exposure (Ctrough or AUC) and efficacy (ORR/DCR). The exposure–efficacy relationship is illustrated as a saturable plateau after sufficient PD-1 target occupancy. The red dashed curve depicts toxicity risk (irAEs), which may increase with higher exposure in susceptible patients and may be modulated by host factors (e.g., genetic background, disease status). The black dashed line represents hypothetical net clinical benefit, which reflects the balance between efficacy and toxicity and may decline at very high exposure. The shaded area indicates a proposed benefit–risk range rather than a validated therapeutic window. The exposure categories (low, moderate, high, very high) and associated efficacy/toxicity levels are for conceptual illustration only. Exact exposure thresholds for Sintilimab in advanced gastric cancer remain to be prospectively defined. Abbreviations: C_trough, trough concentration; AUC, area under the curve; ORR, objective response rate; DCR, disease control rate; irAEs, immune-related adverse events.

Figure 3 illustrates the hypothetical benefit–risk window for Sintilimab based on the conceptual E-R relationship, highlighting the proposed benefit–risk range and emphasizing that these thresholds are schematic and have not been prospectively validated.

Given that most PK and E-R data for Sintilimab in AGC are limited, Table 1 summarizes the evidence landscape for key concepts based on direct Sintilimab/AGC evidence versus extrapolation from other ICIs.

**TABLE 1:** Evidence landscape for key exposure-response concepts in Sintilimab for advanced gastric cancer.

Evidence domain	Direct evidence in Sintilimab/AGC	Evidence from other ICIs	Interpretation for sintilimab in AGC
PK linearity	Limited (phase I data in solid tumors, not AGC-specific)	Strong (nivolumab, pembrolizumab)	Plausible by extrapolation, but AGC-specific validation needed
Time-dependent CL	Insufficient (no dedicated PopPK study in AGC)	Strong (nivolumab, pembrolizumab, avelumab)	Indirect support; likely relevant based on disease dynamics
Tumor burden and CL	Limited (indirect evidence from AGC retrospective studies)	Moderate to strong (pembrolizumab, atezolizumab, avelumab)	Likely relevant; should be considered in PopPK models
Exposure-efficacy plateau	Limited (indirect from PD-1 occupancy data)	Strong (nivolumab, pembrolizumab)	Likely applicable; target saturation drives flat E-R relationship
Exposure-safety	Limited and heterogeneous (real-world data available but confounded)	Mixed (dose-dependent for CTLA-4; less clear for PD-1/PD-L1)	Should be interpreted cautiously; host factors matter
TDM and MIPD	No routine clinical validation (conceptual only)	Emerging (evidence from other ICIs and other drug classes)	Conceptual/translational; prospective studies needed

Abbreviations: AGC, advanced gastric cancer; CL, clearance; E-R, exposure-response; ICIs, immune checkpoint inhibitors; MIPD, model-informed precision dosing; PK, pharmacokinetics; PopPK, population pharmacokinetics; TDM, therapeutic drug monitoring.

## 5 Exposure-safety relationship: the balancing mechanism between immune activation and toxicity risk

### 5.1 Spectrum and clinical characteristics of immune-related adverse reactions

Different from traditional chemotherapy, ICIs enhance anti-tumor immunity by releasing the inhibition of T cells. However, this mechanism may also cause the immune system to attack normal tissues, thus triggering irAEs (Zan et al., 2025). A study involving 125 clinical trials showed that the overall incidence of grade ≥3 treatment-related adverse events (TRAEs) of PD-1/PD-L1 inhibitors was approximately 14%, suggesting that severe adverse reactions cannot be ignored in immunotherapy (Wang Y. et al., 2019). In a retrospective real-world study, among 362 solid tumor patients treated with different PD-1 inhibitors, the standardized incidence of any-grade irAEs in the Sintilimab group (n = 171) was 52.81%, higher than that in the toripalimab group and the pembrolizumab group (Huang et al., 2022). This suggests that there may be differences in the irAE spectrum and incidence among different PD-1 inhibitors. However, this difference may not be fully explained by drug exposure and may also be related to factors such as host background and tumor type. It has been reported that Sintilimab can affect multiple organ systems such as the skin, gastrointestinal tract, endocrine system, nervous system, lungs, kidneys, and heart (Zan et al., 2025; Yang et al., 2022; Ai et al., 2022; Xiong et al., 2024; Wang C. et al., 2024; Lin et al., 2022; Ye et al., 2025; Ren et al., 2024; Xia et al., 2024; Shan et al., 2025; Ji et al., 2022; Li et al., 2024). The extensive organ-specific toxicity spectrum suggests that there may be a complex relationship between drug exposure and the immune activation thresholds of different organs, which is worthy of further mechanism research at the organ level.

### 5.2 Potential mechanisms of drug exposure and immunotoxicity

Severe (≥ grade 3) irAEs are critical safety concerns in ICIs treatment. PD-1/PD-L1 inhibitors enhance T-cell responses by releasing immune negative regulation, thus potentially leading to excessive immune activation and off-target tissue damage (Martins et al., 2019/09). In NSCLC patients receiving ICIs treatment, the quantitative relationship between the incidence of irAEs and drug exposure has been validated in a model-based meta-analysis. This analysis pooled trial-level data from 81 clinical studies and 129 cohorts. The results showed that when CTLA-4 inhibitors were used as monotherapy or in combination with PD-1/PD-L1 inhibitors, the incidence of irAEs exhibited a significant dose-dependence (Zhang et al., 2022). Different from the possible “plateau effect” in the efficacy relationship, the exposure-safety relationship may be closer to linear in some situations, meaning that a higher level of drug exposure may correspond to a higher frequency or more severe irAEs.

Real-world studies have shown that although the incidence of any-grade irAEs of Sintilimab is relatively high, its incidence of grade ≥3 irAEs (7.12%) is lower than that of pembrolizumab (13.21%), especially in grade ≥3 pneumonia (Huang et al., 2022). This suggests that the assumption that “higher exposure necessarily leads to higher toxicity” does not always hold true. In addition to the differences in the inherent toxicity profiles of drugs, host factors may also play an important role. Previous studies have indicated that the immunogenetic background is an important determinant of irAEs susceptibility. In patients with specific human leukocyte antigen (HLA) genotypes or immune checkpoint-related gene polymorphisms, ICIs may induce a stronger immune response (Abdel-Wahab et al., 2021; Sasaki et al., 2024; Abed et al., 2022; Akturk et al., 2022; Yano et al., 2020; Hasan Ali et al., 2019; Cappelli et al., 2019; Kośmider et al., 2025). Therefore, understanding the risk of irAEs cannot rely solely on the level of exposure but also requires comprehensive consideration of genetic susceptibility and organ-specific vulnerability.

In addition to HLA typing, factors such as polygenic risk score (PRS), single nucleotide polymorphism (SNP), and microRNA (miR) also provide new perspectives for the prediction of irAEs (Khan et al., 2020; Refae et al., 2020; Bins et al., 2018; Marschner et al., 2020). Existing studies have shown that PRS is associated with the incidence and treatment outcomes of skin irAEs, suggesting an immunogenetic predisposition in patients’ responses to ICIs treatment (Khan et al., 2020). Specific SNP loci in immune-related genes (e.g., UNG, IFNW1, CTLA-4, PD-L1, and IFNL4) have also been confirmed to be associated with the occurrence of irAEs (Refae et al., 2020). Moreover, the expression level of microRNA-146a (miR-146a) and its related SNPs are related to the severity of irAEs (Marschner et al., 2020). Overall, the occurrence of irAEs is the result of the combined action of drug exposure and immunogenetic background, with obvious individual heterogeneity and organ specificity.

### 5.3 Regulation of toxicity risks by host factors and biomarkers

Previous studies have shown that patients with irAEs tend to have better PFS, OS, and ORR (Das and Johnson, 2019) than those without toxic reactions, suggesting that irAEs are associated with stronger immune activation and better efficacy to a certain extent. However, while irAEs bring potential efficacy benefits, they may also lead to treatment interruption or even termination, thus affecting the overall treatment continuity (Kośmider et al., 2025). A Finnish retrospective real-world cohort study showed that among 317 adult patients with inoperable locally advanced or metastatic cancer, 50 (17%) discontinued ICIs due to irAEs, with pneumonia, hepatitis, and colitis being the most common (Virtanen et al., 2024). In addition, some severe irAEs can progress to fatal events, especially immune-related pneumonia, myocarditis, and neurological toxicity. Although the incidence is low, the case fatality rate is relatively high (Mahmood et al., 2018; Wang et al., 2018; Moslehi et al., 2018). Therefore, constructing a prediction model that takes into account both efficacy benefits and toxicity risks has important clinical value.

Cytokines, C-X-C motif chemokine ligands (CXCL), and soluble immune molecules play a central role in the balance of immune responses and are potential predictive biomarkers for irAEs (Bracamonte-Baran and Kim, 2024). Studies have shown that the baseline levels and dynamic changes of various inflammation-related cytokines (such as IL-6, TNF-α, IL-8, and CXCL9/10) are closely related to the risk of irAE occurrence (Les et al., 2023; Chennamadhavuni et al., 2022; Ke et al., 2020; Wang et al., 2022b; Tarhini et al., 2015; Khan et al., 2019; Nuñez et al., 2023; Zhang et al., 2024; Chen et al., 2021; Wang et al., 2021; Kang et al., 2021; Yu et al., 2022; Lim et al., 2019). Among them, the levels of IL-6 and some CXCL family factors increase significantly in the early stage after treatment, which can serve as early warning signals for the occurrence of irAEs; specific cytokine profiles may also correspond to different types of organ toxicities, such as immune-related colitis and autoimmune myositis (Chennamadhavuni et al., 2022; Ke et al., 2020; Wang et al., 2022b). Mechanistically, these factors not only participate in the amplification of the inflammatory cascade but may also link the intensity of drug exposure to systemic immune toxicity, thus having high value for translational research.

In addition to cytokines, peripheral blood cell parameters and immune cell subsets are also important sources of information for predicting irAEs. Lymphocyte count, neutrophil/lymphocyte ratio (NLR), platelet/lymphocyte ratio (PLR), and C-reactive protein (CRP) in routine blood tests have been confirmed in multiple studies to be associated with the risk of irAE occurrence (Bracamonte-Baran and Kim, 2024; Diehl et al., 2017; Pavan et al., 2019; Peng et al., 2020; Eun et al., 2019; Valpione et al., 2018; Xu et al., 2025). More detailed immunophenotypic analysis further suggests that different T-cell and B-cell subsets are differentially associated with different types and severities of irAEs (Bukhari et al., 2023; Benesova et al., 2022; Miao et al., 2022; Damuzzo et al., 2016; Oh et al., 2017; Das et al., 2018). These results indicate that irAEs are not driven by a single inflammatory pathway but are a comprehensive process involving innate immunity, adaptive immunity, and cellular population remodeling. Combining the analysis of peripheral immune cell profiles with pharmacokinetic parameters may help improve the accuracy of toxicity prediction.

Pre-existing autoantibodies are also important indicators for evaluating patients’ autoimmune predisposition and are closely related to the mechanism of autoantibody-mediated tissue damage (Taylor et al., 2022; Tahir et al., 2019). Among them, anti-thyroid peroxidase (TPO) and anti-thyroglobulin (Tg) have sufficient evidence in predicting thyroid dysfunction (Tang et al., 2022; Aung et al., 2023; Les et al., 2021; Toi et al., 2019; Kimbara et al., 2018; Maekura et al., 2017; Kurimoto et al., 2020). In contrast, the predictive value of anti-nuclear antibody (ANA) remains controversial, and the results of different studies are inconsistent (Tang et al., 2022; Aung et al., 2023; Sakakida et al., 2020; Barth et al., 2022). In addition, some non-classical autoantibodies, such as anti-striated muscle antibodies, anti-type XVII collagen IgG (anti-BP180) antibodies, and autoantibodies associated with hypophysitis or pneumonia, also suggest that the autoimmune background may determine the predisposition to specific organ irAEs (Tahir et al., 2019; Ono et al., 2022; Bilen et al., 2016; Jeyakumar et al., 2020; Hasan Ali et al., 2020).

As an emerging class of biomarkers, microbiome dysbiosis reflects the regulatory effect of the gut microbial ecosystem on the host immune system (Seton-Rogers, 2021; Zhang et al., 2023; Xu et al., 2019; Belkaid and Hand, 2014). Studies have found that a relatively high abundance of Bacteroidetes may be associated with a reduced risk of colitis irAE, while a microbiome rich in Firmicutes is associated with a higher incidence of colitis in some studies (Zhang et al., 2023; Dubin et al., 2016). In addition, certain specific genera or species are associated with organ-specific irAEs such as arthritis/arthralgia, hypothyroidism, and pneumonia (McCulloch et al., 2022). These results suggest that the gut microbiome may influence the risk of ICIs-related toxicity by shaping the systemic immune response, and has potential value for prediction and intervention.

In summary, irAEs exhibit a double-edged sword characteristic of “coexistence of efficacy correlation and toxicity risk”. Diverse biomarkers together form a complex toxicity prediction network, providing a basis for combining pharmacokinetic parameters with host factors to establish a multi-dimensional risk stratification system. In the future, large-scale, prospective pharmacokinetic and pharmacodynamic studies are still needed to integrate information on immune genetic background, inflammatory factors, and dynamic biomarkers to achieve accurate prediction and individualized management of severe irAEs.

## 6 Therapeutic drug monitoring: from theoretical foundations to clinical translation challenges

### 6.1 Theoretical basis and applicability of therapeutic drug monitoring in immunotherapy

TDM is an important strategy for achieving individualized and precise medication. It assesses the drug exposure level in patients by measuring the blood drug concentration at specific time points. Although ICIs have widely adopted the fixed-dose administration mode, there are still moderate to high inter-individual differences in their *in vivo* exposure (Elassaiss-Schaap et al., 2017; Bajaj et al., 2017a). Previous studies on Nivolumab and Pembrolizumab have shown that within the clinically recommended dosage range, their exposure-efficacy curve is generally flat, and the exposure-safety relationship does not show a clear dose-dependent enhancement (Liu et al., 2017; Agrawal et al., 2016; Wang et al., 2017; Chatterjee et al., 2016). Therefore, TDM in the field of ICIs is more suitable to be regarded as a tool for explaining inter-individual differences and assisting in optimizing treatment strategies, rather than the simple model of “directly adjusting the dosage based on concentration” in the traditional sense.

The prerequisite for implementing TDM is the availability of a validated and standardized bioanalytical method. Therefore, the application of TDM for ICIs depends on sensitive and specific detection methods (Oude Munnink et al., 2016). Currently, for the quantitative analysis of macromolecular protein drugs such as monoclonal antibodies, commonly used techniques include enzyme-linked immunosorbent assay (ELISA), electrochemiluminescence immunoassay (ECLIA), and liquid chromatography-tandem mass spectrometry (LC-MS/MS) (Centanni et al., 2019). Among them, ELISA is based on the specific binding of antigen-antibody. It has the advantages of simple operation and low cost, and is one of the most widely used platforms in clinical and basic research, such as for detecting biomarkers like soluble PD-1 (sPD-1) and soluble PD-L1 (sPD-L1) (Kurosaki et al., 2023). ECLIA has higher sensitivity and a wider linear detection range, and is suitable for high-throughput automated detection. LC-MS/MS shows unique advantages in scenarios requiring high-precision quantification or complex matrix analysis involving metabolites, and can effectively distinguish molecules with similar structures. For example, the plasma concentrations of ripretinib and its multiple metabolites have been accurately quantified, but its application in routine clinical research is still limited (Zhang et al., 2025). Different methods have their own characteristics, and in practical applications, factors such as detection sensitivity, specificity, throughput, and cost need to be comprehensively considered for selection.

Moreover, theoretically, the dose optimization strategy based on drug exposure is expected to reduce unnecessary drug use while ensuring efficacy, thus providing a potential pharmacoeconomic basis for the application of TDM in immunotherapy. For Sintilimab, although dedicated TDM studies in patients with AGC are currently lacking, the observed inter-individual pharmacokinetic variability and the potential for time-dependent clearance suggest that TDM may hold exploratory value in specific scenarios, such as when ADA-mediated accelerated clearance is suspected or in patients with extreme body weight, although prospective validation is still required.

### 6.2 Research status and controversies in therapeutic drug monitoring of immune checkpoint inhibitors

TDM holds significant research value in ICIs treatment. Due to the substantial pharmacokinetic differences among patients, even under the standard dosing regimen, there may still be considerable fluctuations in drug exposure among individuals. This difference is considered one of the key pharmacological bases for the heterogeneity of clinical efficacy and toxic reactions. Taking the phase I study of Sintilimab combined with anti-LAG-3 antibody IBI110 in the treatment of advanced solid tumors (including gastric cancer) as an example, the difference in drug exposure and its potential clinical impact may be jointly related to factors such as the patient’s pathophysiological state, characteristics of the TME, and immunogenicity (Mao et al., 2024). These factors affect the drug distribution and clearance processes, ultimately leading to differences in blood drug concentrations, which further influence the efficacy and safety outcomes.

Mechanistically, patients with AGC often present with characteristics such as low albumin levels, systemic inflammatory status, and high tumor burden. These factors may increase the clearance rate and reduce the exposure level of ICIs, leaving some patients in a relatively low-exposure state. Ctrough,ss, as a key indicator reflecting the lowest systemic exposure level, is highly operable in real-world cohorts. It can be used to identify patients with potential insufficient exposure and provide a reference for subsequent treatment adjustment. On the other hand, when severe irAEs occur or the formation of ADA is suspected, blood drug concentration detection can also serve as an auxiliary diagnostic tool. Existing studies have shown that the ADA incidence rate of Nivolumab is approximately 11.2%, which can increase the clearance rate by about 14%; the ADA incidence rate of Pembrolizumab is approximately 0.7%–2.5% (Li et al., 2017; Bajaj et al., 2017a; Vugt et al., 2016). In contrast, for Sintilimab, a fully humanized antibody, the ADA incidence rate and its impact on the clearance rate still need further clarification.

In addition, the current ICIs administration strategies mainly include two modes: fixed-dose and body weight-based dosing. However, there is still a controversy over which one is superior. Although fixed-dose administration can simplify the process and reduce operational complexity, it remains undetermined whether it can effectively reduce the inter-patient exposure differences, especially when there are significant individual pharmacokinetic differences (Centanni et al., 2019). Pharmacokinetic analyses in registered clinical trials are usually based on strictly screened populations, while in the real-world environment, the exposure range of patients may be wider, even exceeding that observed in registered trials (Centanni et al., 2019). Taking Ipilimumab as an example, the correlation between its exposure and efficacy suggests that reducing inter-patient exposure differences has potential value; although the body weight-related dosing strategy can help optimize exposure to some extent, it can only explain part of the individual differences (Feng et al., 2014). Therefore, in the future, it is still necessary to combine strategies such as TDM to explore how to achieve more reasonable exposure control at the individual level.

Notably, although ICIs generally lack clear dose-limiting toxicities, it is still of great significance to establish a reasonable exposure range within the efficacy range. For example, the exposure-response curve of Nivolumab reaches a saturation state below the recommended marketing dose (Agrawal et al., 2016), suggesting that it is theoretically feasible to appropriately reduce the dose or extend the dosing interval without affecting the efficacy (Ratain and Goldstein, 2018), indicating that TDM has potential value in optimizing the dosing strategy and reducing the treatment cost.

### 6.3 Potential application value and implementation challenges of therapeutic drug monitoring for sintilimab

There are significant inter-individual pharmacokinetic differences in Sintilimab. Theoretically, insufficient exposure may lead to poor efficacy, while excessive exposure may increase the risk of toxicity (Jiao et al., 2021). Therefore, TDM has potential application value in Sintilimab treatment. The optimization goal of immunotherapy is not only to improve the anti-tumor efficacy but also to minimize the risk of treatment interruption and death caused by severe irAEs. Thus, it is particularly important to construct an exposure-response framework that takes both efficacy and safety into account.

The ultimate goal of exposure-efficacy and exposure-safety analyses is to define the “therapeutic window” of a drug, which is the exposure range that maximizes efficacy while keeping the toxicity risk within an acceptable level (Ji and Sy, 2024).

By integrating relevant models and conducting clinical trial simulations, the proportions of patients falling within, below, or above this therapeutic window under different dosing regimens (dose, frequency) can be evaluated, thus providing a quantitative basis for optimizing dosing strategies.

In this process, TDM can serve as a dynamic monitoring tool to identify differences in drug exposure and assist in individualized dose adjustment, enabling patients’ Sintilimab exposure to approach the optimal range as much as possible. Previous studies have shown that TDM has more application advantages when there are large inter-individual exposure differences, relatively stable intra-individual variations, a clear association between exposure and effect, and a reliable detection method (Oude Munnink et al., 2016). Moreover, from a pharmacoeconomic perspective, exposure-based precise dose adjustment is also expected to reduce the additional medical costs caused by treatment failure and severe toxicity (Kantasiripitak et al., 2020).

However, the current prospective clinical studies on TDM of Sintilimab are still very limited. The specific exposure-effect threshold, the optimal monitoring time point, and the TDM-based dose adjustment algorithm all need to be further explored and validated in patients with AGC before it can be translated from a research tool into routine clinical practice.

## 7 Precision treatment strategies and future directions: towards model-guided individualized immunotherapy

### 7.1 Model-guided precision dosing based on pharmacokinetics

MIPD is an optimized dosing strategy that combines quantitative pharmacology models with individual patient characteristics. Its core lies in achieving dynamic dose adjustment by predicting individual drug exposure levels, thereby maximizing efficacy while reducing the risk of toxicity (Darwich et al., 2021; Minichmayr et al., 2024). Different from traditional TDM, which mainly relies on empirical concentration ranges, MIPD achieves more precise dosing decisions by integrating PopPK models, PK-PD relationships, and individual covariates such as body weight, albumin level, tumor burden, and immune status (Minichmayr et al., 2024). Methodologically, MIPD is usually based on the nonlinear mixed-effects model (NLME). The PopPK model is used to characterize the variations between the population and individuals, and then the PK-PD model is combined to associate drug exposure with efficacy or toxicity endpoints (such as ORR or irAEs). On this basis, the Bayesian estimation method can update parameters by combining prior model information with individual real-time monitoring data (such as Ctrough or AUC), thus achieving dose optimization at the individual level (Kantasiripitak et al., 2020; Darwich et al., 2021). With the improvement of computing power, machine learning and reinforcement learning methods have also been gradually introduced into the MIPD framework for dealing with complex nonlinear relationships and high-dimensional data integration (Minichmayr et al., 2024).

It should be noted that the unique time-dependent clearance characteristics of ICIs pose higher requirements for MIPD. This phenomenon was first systematically identified in Nivolumab, and subsequently, a PopPK model incorporating time-varying CL was developed. The model shows that its CL can gradually decrease during the treatment process, with a maximum reduction of approximately 25% of the baseline value (Bajaj et al., 2017a). This indicates that if the model fails to incorporate time-varying clearance, the true E-R relationship may be misjudged. To address the bias in E-R analysis caused by time-dependent clearance, researchers have proposed various modeling and research strategies, such as the Cox proportional hazards (CPH) model combined with case-matching analysis, the tumor growth inhibition–overall survival (TGI-OS) joint modeling, and the design of multi-dose level randomized controlled trials (Kawakatsu et al., 2021). The common goal of these methods is to distinguish the interactive effects of drug effects, disease progression, and prognostic factors as much as possible, so as to more reliably identify the true exposure-response relationship. For this reason, traditional fixed-dose or body weight-based dosing strategies often fail to fully reflect individual differences, while model-based dosing optimization is more likely to achieve precise exposure control.

Currently, MIPD has been successfully applied in drugs such as vancomycin and tamoxifen, significantly improving target exposure attainment rate and reducing the toxicity risk (Hughes et al., 2021; Mueller-Schoell et al., 2021). However, its clinical application in tumor immunotherapy is still in the exploratory stage. In clinical practice, MIPD usually follows a closed-loop process of “model prediction-data feedback-dose adjustment”. First, the initial dose is designed based on the PopPK model. Subsequently, by monitoring drug concentrations or relevant biomarker data such as Ctrough, AUC, IL-6, or immune cell subsets, the Bayesian method is used to update individual parameters, and finally, an individualized dosing regimen is output (Pérez-Blanco and Lanao, 2022). This dynamic optimization process can reflect the patient’s disease state and pharmacokinetic changes in real-time, thereby improving the accuracy and safety of treatment.

Although MIPD has significant theoretical advantages, its clinical promotion is also limited by issues such as insufficient external validation of the model, limited support from real-world data, lack of prospective randomized controlled studies to verify its clinical benefits, high computational complexity, high implementation costs, and poor clinical operability (Darwich et al., 2017). In the future, multi-center prospective studies and standardized model construction strategies are needed to promote the clinical translation and application of MIPD in the field of immunotherapy.

**FIGURE 4:**
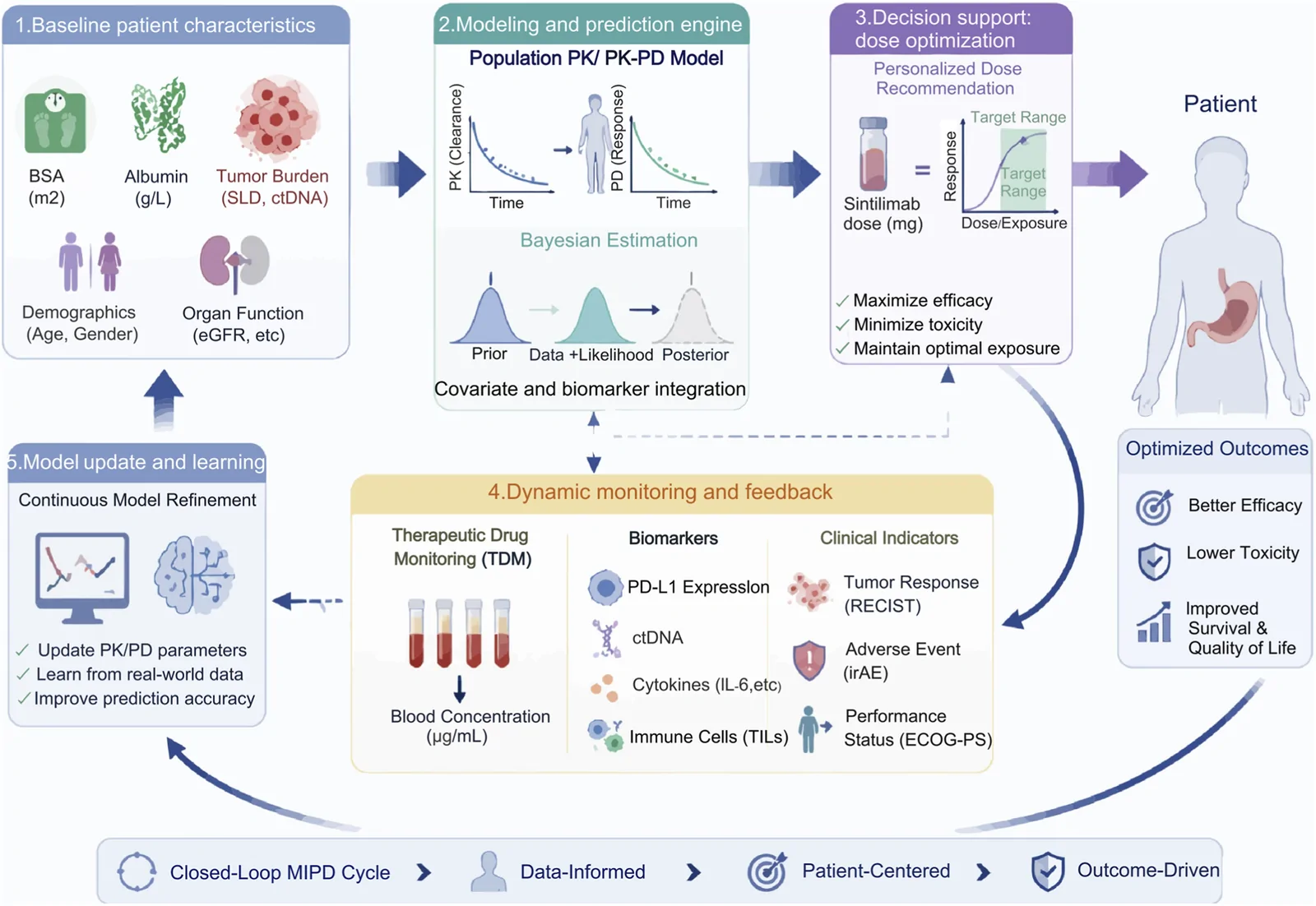
Model-informed precision dosing (MIPD) decision loop for Sintilimab. The flowchart shows integration of TDM and PK/PD modeling with individual patient E-R data. Patients’ drug exposure is compared with the defined therapeutic window, and dose adjustments are made iteratively to optimize efficacy while minimizing the risk of irAEs.

Figure 4 illustrates the MIPD decision loop for Sintilimab, integrating TDM, PK-PD modeling, and patient-specific E-R data to guide individualized dose adjustments.

### 7.2 Multidimensional biomarker-driven risk stratification strategies

As previously mentioned, single biomarkers have obvious limitations in predicting the efficacy of ICIs and irAEs. The outcomes of ICIs treatment are not driven by a single factor but are jointly regulated by multidimensional factors such as drug exposure (e.g., Ctrough, AUC), host immunogenetic background (e.g., HLA polymorphism, ADA, and genetic variations related to immune checkpoints), systemic inflammatory status (e.g., NLR, CRP, IL-6), peripheral immune cell subsets, and tumor-related characteristics (e.g., PD-L1 expression, TMB, and TME composition) (Les et al., 2023; Wang J. et al., 2024; Trontzas et al., 2025). Therefore, in recent years, research has gradually shifted from single-index analysis to risk stratification models integrating multidimensional biomarkers to achieve a comprehensive assessment of efficacy benefits and toxicity risks. Mechanistically, the outcomes related to ICIs essentially stem from the dynamic balance among T cell activation driven by drug exposure, inflammation amplification, and remodeling of the TME. Models integrating pharmacokinetic parameters, host immunogenetic characteristics, inflammatory factor networks, and TME information are more conducive to revealing the synergistic and antagonistic effects among different biological levels and enhancing the ability to simultaneously predict efficacy and severe irAEs.

With the development of multi-omics technologies, the integrated analysis based on data from transcriptomics, proteomics, metabolomics, and microbiome has provided new possibilities for constructing high-dimensional and dynamic prediction models (Bodein et al., 2022; Ouyang et al., 2023). In particular, combining multi-omics data with PopPK and PK-PD models is expected to achieve the joint prediction of individual drug exposure, immune activation status, and toxicity risks. Meanwhile, the advantages of machine learning and artificial intelligence methods in handling high-dimensional and complex data also provide important technical support for the integration of multi-dimensional biomarkers, making it possible to transform the prediction from “static indicator prediction” to “dynamic risk assessment” (Miao et al., 2024; Mu et al., 2026). Through multi-dimensional data in the early stage of treatment, including baseline characteristics, initial blood drug concentration, and omics features, the model is expected to more accurately predict the long-term efficacy of Sintilimab and the risk of specific toxicities such as immune-related pneumonia, hepatitis, and skin reactions in patients with advanced gastric cancer (Zhu et al., 2025; Qin et al., 2025; Yan et al., 2022). This integrated analysis framework provides a theoretical basis for achieving true precision medicine, that is, formulating prospective treatment strategies according to patients’ individual biological characteristics and drug exposure, rather than relying on *post hoc* empirical adjustments.

In conclusion, the efficacy and safety of ICIs treatment essentially stem from the dynamic balance among “drug exposure-host immunity-inflammatory response”. The risk stratification model integrating multi-dimensional biomarkers can achieve information fusion within this complex network, thereby enhancing the predictive ability for efficacy and irAEs. In the future, constructing a comprehensive prediction system for clinical decision-making support by combining the PopPK model, dynamic biomarker monitoring, and machine learning algorithms will promote the development of MIPD and individualized immunotherapy.

### 7.3 Construction of dynamic exposure-response model and future research directions

Although Sintilimab combined with chemotherapy has shown clear clinical benefits in AGC, the current evidence system still mainly remains at the stage of “population-level efficacy evaluation” and faces various challenges in the process of transformation to “individualized precision treatment”. First, at the level of quantitative pharmacology, there are still obvious gaps in the PopPK and E-R models of Sintilimab in gastric cancer patients (Shang et al., 2022). Although key studies such as ORIENT-16 have accumulated relatively rich pharmacokinetic data, the relevant model analyses have not been fully disclosed, making it difficult to systematically characterize the full-chain relationship of dose-exposure-efficacy/toxicity. A critical limitation is that in the ORIENT-16 trial, the contribution of sintilimab exposure to overall efficacy cannot be easily isolated from the effect of concurrent chemotherapy and baseline prognostic factors. This confounding should be carefully considered when interpreting the E-R relationship. Second, there is a lack of sufficient quantitative support for the optimization of the dosing strategy. The weight-stratified combined fixed-dose regimen used in the ORIENT-16 study takes into account both pharmacological rationality and clinical operability, but the exposure equivalence in different weight populations still needs systematic verification (Xu et al., 2023; Xu et al., 2021; Shchelokov and Demin, 2020). Third, at the biomarker level, in addition to PD-L1 CPS, the specific values of potential predictive indicators such as MSI-H, TMB, cytokines, and TGF-β in the combined treatment of Sintilimab still lack systematic verification (Zhao et al., 2025). In addition, the pharmacokinetic characteristics and E-R relationships in special populations such as those with low body weight, the elderly, and those with abnormal liver and kidney functions are not clear. Moreover, the existing key studies are mainly based on Asian populations, and the extrapolation of the results to different ethnic populations still needs further evaluation (Liu et al., 2022).

In light of the aforementioned challenges, future research can focus on three aspects: “model construction-mechanism integration-clinical translation”. On one hand, large-scale clinical trial data should be prioritized to construct and validate PopPK and E-R models specific to gastric cancer, aiming to quantitatively describe individual exposure differences and their clinical implications. On the other hand, during the model construction process, multi-dimensional biomarkers (including immunogenetic background, inflammatory indicators, and TME characteristics) should be integrated to explore the interactions between drug exposure and host factors, thereby improving the accuracy of efficacy and toxicity prediction. Based on this, optimizing dosing strategies through model simulation, such as evaluating the applicability of fixed doses and exploring long-interval dosing regimens, and validating their applicability in a broad population with real-world evidence (RWE), will contribute to the translation of research findings into clinical practice.

In addition, with the development of multi-omics technologies and artificial intelligence methods, immunotherapy research is moving from traditional single-index analysis to integrated modeling driven by multi-dimensional data. In this context, Quantitative Systems Pharmacology (QSP) models, which integrate drug PK/PD with tumor-immune dynamics at a systems level, have been applied to characterize inter-individual variability in anti-PD-(L)1 efficacy and to predict clinical responses to checkpoint blockade (Leete et al., 2022). Such approaches may eventually provide a mechanistic scaffold for predicting individual responses to Sintilimab and identifying optimal dosing strategies, although their application specifically in AGC remains to be established. In the future, a high-dimensional dynamic prediction system can be constructed by integrating multi-level data such as transcriptomics, proteomics, metabolomics, and immunomics, and combining PopPK and PK-PD models to achieve a joint assessment of drug exposure, immune activation status, and toxicity risks. Meanwhile, the advantages of machine learning and reinforcement learning methods in handling complex non-linear relationships are also expected to further enhance the application value of the model in individualized efficacy prediction and dosing optimization. The above multi-dimensional integration strategy will provide key support for MIPD and promote immunotherapy to move from experience-driven to data-driven precision medicine mode.

**FIGURE 5:**
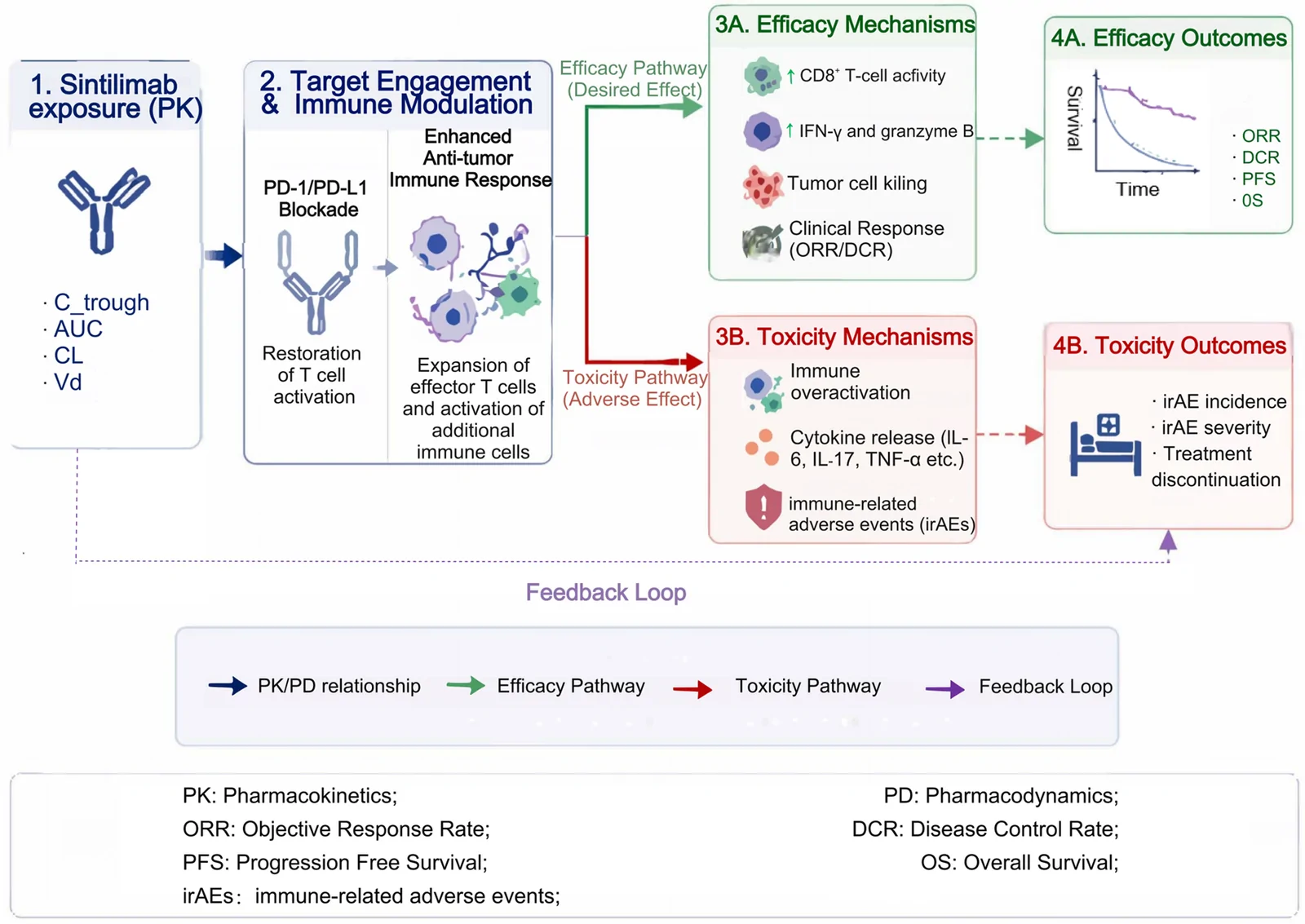
Conceptual exposure–response (E-R) flowchart of Sintilimab. The diagram integrates Sintilimab pharmacokinetics (PK; C_trough, AUC, CL, Vd) with PD-1/PD-L1 blockade and subsequent restoration of T cell activation. In this framework, PD-1 is expressed on T cells, whereas PD-L1 is expressed on tumor cells or antigen-presenting cells. From this point, two diverging pathways are depicted: the efficacy pathway (desired effect), involving CD8^+^ T-cell activity, IFN-γ and granzyme B release, and tumor cell killing, leading to clinical response (ORR, DCR, PFS, and OS); and the toxicity pathway (adverse effect), driven by immune overactivation and cytokine release (IL-6, IL-17, TNF-α, etc.), leading to immune-related adverse events (irAEs). Feedback loops at the bottom indicate bidirectional interactions between PK/PD and both efficacy and toxicity outcomes. Specifically, clinical response, tumor burden reduction, changes in inflammatory status, and treatment discontinuation may feed back into PK/PD dynamics, thereby influencing subsequent drug exposure and response. This figure represents a conceptual PK–PD framework and is not a validated exposure–response model for Sintilimab. Abbreviations: PK, pharmacokinetics; C_trough, trough concentration; AUC, area under the curve; CL, clearance; Vd, volume of distribution; ORR, objective response rate; DCR, disease control rate; PFS, progression-free survival; OS, overall survival; irAEs, immune-related adverse events; PD, pharmacodynamics.

Figure 5 illustrates the mechanistic E-R framework of Sintilimab, highlighting the interplay between PK, PD-1/PD-L1 blockade, T-cell activation, and downstream efficacy and toxicity pathways.

## 8 Conclusion

In conclusion, Sintilimab has demonstrated clear clinical benefits in the treatment of AGC. Its efficacy and safety essentially stem from the dynamic balance among drug exposure, host immune status, and inflammatory response. However, the current quantitative understanding of this balance system still mainly remains at the population level. The mechanisms and prediction of individual exposure differences and their impacts on efficacy and irAEs still need to be further elucidated. This review conceptualizes a dynamic exposure-response framework for Sintilimab in AGC by integrating time-dependent clearance, disease-state confounding, biomarker modulation, TDM, and MIPD. The framework provides a conceptual basis for MIPD, with the goal of optimizing individualized therapy and improving the benefit-risk balance for patients with AGC. In the future, with the development of PopPK, E-R, and PK-PD models, and combined with the integrated analysis of multi-dimensional biomarkers, multi-omics data, and artificial intelligence methods, it may eventually contribute to the development of precision immunotherapy, but this requires prospective validation and remains a future aspiration rather than a current clinical reality. On this basis, promoting the clinical translation of MIPD will provide crucial support for optimizing immunotherapy strategies, improving efficacy, and reducing the risk of toxicity, ultimately facilitating the continuous optimization of personalized treatment for patients with AGC.
